# Positive association between different triglyceride-glucose index-related indicators and overactive bladder: Results from NHANES (2013–2020)

**DOI:** 10.1097/MD.0000000000047949

**Published:** 2026-03-13

**Authors:** Yong Luo, Shurui Li, Yi Lu, Dejian Hou, Jifang Yang

**Affiliations:** a Department of Nephrology, Shehong Municipal Hospital of Traditional Chinese Medicine, Sichuan, China; b Department of Orthopedics, The Traditional Chinese Medicine Hospital of Longquanyi, Sichuan, China.

**Keywords:** insulin resistance, NHANES, overactive bladder, TyG-BMI, TyG-WC, TyG-WHtR

## Abstract

The triglyceride-glucose (TyG) index is a promising new biomarker for insulin resistance, but its relationship with the risk of overactive bladder (OAB) has not yet been studied. This nationwide study aims to investigate the association between TyG-related indices and the risk of OAB. In this study, we analyzed a dataset from the 2013 to 2020 National Health and Nutrition Examination Survey that was nationally representative. Only participants who had complete data on TyG-related indices and OAB were selected for inclusion. Through weighted multivariable logistic regression, we assessed the association between TyG-related indices and OAB, making adjustments for numerous confounders. Additionally, comprehensive subgroup analyses were undertaken to confirm the validity of the findings. Sensitivity analyses were performed by excluding participants with diabetes, hypertension, or extreme laboratory values to assess the robustness of the results. The discriminative ability of TyG-related indices for identifying OAB was further explored via receiver operating characteristic (ROC) curves. The study analyzed 7841 participants, among whom 1962 were diagnosed with OAB. In the fully adjusted model, triglyceride-glucose body mass index, triglyceride-glucose waist circumference, and triglyceride-glucose waist-to-height ratio (TyG-WHtR) in quartiles 4 and 3 compared with quartile 1 were significantly associated with OAB. Among these, TyG-WHtR showed a more pronounced relationship with OAB (TyG-WHtR model 3: Q4 odds ratio [95% confidence interval] = 2.138 [1.635–2.797]). The ROC analysis also indicated that TyG-WHtR had a stronger discriminative ability for identifying OAB: 0.639 (95% confidence interval: 0.625–0.653). Sensitivity analyses confirmed the robustness of these associations across different analytical conditions. A linear association between TyG-related indices and OAB was observed within the restricted cubic spline regression model limits. Furthermore, both subgroup and ROC analyses demonstrated that males were more likely to be affected by TyG-related indices than females. The findings of this study establish a clear association between the triglyceride-glucose body mass index, triglyceride-glucose waist circumference, and TyG-WHtR indices and OAB. These results suggest that TyG-related indices may serve as potential indicators warranting further investigation in OAB research.

## 1. Introduction

Overactive bladder (OAB) is a prevalent medical condition that gravely compromises quality of life. It presents with an immediate, uncontrollable compulsion to void urine, frequently accompanied by increased urination frequency and nocturia. In the US demographic, OAB prevalence is virtually identical among men (16.0%) and women (16.9%), and escalates with age.^[1]^ Healthcare expenditures for those afflicted with OAB are substantially more than 2.5 times than those for individuals without this condition.^[2,3]^ Statistically, OAB incurs billions of dollars in annual medical expenses in the United States,^[4]^ indicating a substantial economic burden on both patients and the society. Despite its prevalence, the etiology of OAB remains unclear. Some studies suggest that OAB may be associated with obesity, diabetes, socioeconomic status, and unhealthy lifestyles.^[5,6]^

Recent studies have linked metabolic syndrome (MS) and insulin resistance (IR) to OAB, with affected patients frequently exhibiting glucose and lipid metabolism abnormalities.^[7–10]^ The triglyceride-glucose (TyG) index has emerged as a practical surrogate marker for IR, offering advantages over traditional methods such as the hyperinsulinemic-euglycemic clamp and homeostatic model assessment of IR, which are complex and time-intensive.^[11]^ Refined indices incorporating anthropometric measurements – triglyceride-glucose body mass index (TyG-BMI),^[12]^ waist-to-height ratio (TyG-WHtR),^[13]^ and waist circumference (TyG-WC)^[14]^ – have been developed to better characterize IR severity. However, existing literature does not address the association between various TyG-related indices and OAB.

Consequently, the objective of this study is to examine the possible associations between TyG-related indices and OAB utilizing a nationally representative US adult sample from the National Health and Nutrition Examination Survey (NHANES), yielding important insights into how IR relates to OAB.

## 2. Methods

### 2.1. Study participants in NHANES

**Figure 1.**
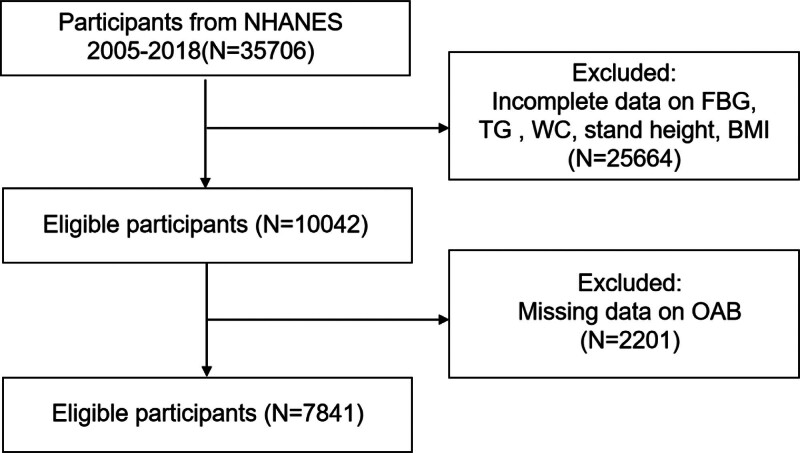
Flowchart of participants selection. BMI = body mass index, FBG = fasting blood glucose, NHANES = National Health and Nutrition Examination Survey, OAB = overactive bladder, TG = triglycerides, WC = waist circumference.

The research involved a cross-sectional study utilizing data from NHANES. Every data collection protocol associated with NHANES was endorsed by the National Center for Health Statistics Research Ethics Review Committee, and informed consent was secured from each participant. Due to the inability to comprehensively evaluate all participants, only those meeting the predefined criteria were included in our study. We excluded individuals with incomplete data on OAB and TyG-related indices. After applying these criteria, 7841 participants were deemed eligible for analysis. Figure 1 illustrates the inclusion and exclusion criteria.

### 2.2. OAB

In this study, OAB was defined as an Overactive Bladder Symptom Score (OABSS) ≥ 3, consistent with previous epidemiological studies using NHANES data.^[15]^ The International Continence Society (ICS) identifies urgency urinary incontinence and nocturia as the primary features of OAB, which constitute the foundation of its assessment. Trained researchers obtained all necessary diagnostic information for OAB from questionnaires during face-to-face interviews. Participants were surveyed with 2 questions to assess the severity of urgency urinary incontinence: “In the past 12 months, have you had any incidents of urine leakage or loss of bladder control, even minimal, accompanied by a sudden urge to urinate and a difficulty in reaching the toilet promptly?” and “How often did such incidents occur?” To measure nocturia, the following question was administered: “Over the last 30 days, from when you went to bed at night until you woke up in the morning, how many times did you usually need to urinate?” Researchers then employed the OABSS to determine the severity of OAB based on these questionnaire responses. It should be noted that this scoring-based definition differs from the standard ICS clinical definition, which characterizes OAB by the presence of urinary urgency, usually accompanied by frequency and nocturia, without a specific numerical threshold.

### 2.3. Equation for TyG-related metrics calculation

Calculation of the TyG index is based on the formula ln([fasting triglycerides [mg/dL] × fasting glucose [mg/dL]]/2).^[16]^ TyG-BMI is derived by multiplying the TyG index with BMI.^[17]^ The TyG-WC results from the TyG index multiplied by the waist circumference.^[18]^ WHtR is defined as waist circumference divided by standing height, and TyG-WHtR results from multiplying the TyG index by WHtR.^[13]^

### 2.4. Covariates assessment

For this study, other covariates were obtained through standard questionnaires and physical examinations. These included age, gender, race/ethnicity, marital status, education level, household income, smoking status, hypertension, and diabetes status, with the goal of minimizing potential confounding biases. Demographic questionnaires self-reported by participants provided detailed information on age, gender, race/ethnicity (non-Hispanic White, non-Hispanic Black, Mexican American, Other), educational attainment (ranging from less than high school to more than high school), marital status (including categories such as married, cohabiting, widowed, divorced, separated, or never married), and the poverty-income ratio (PIR). PIR was defined in 3 strata: <1.3, 1.3 to 3.0, and >3.0. Inquiry into smoking status was conducted via the question, “Have you smoked at least 100 cigarettes in your entire life?” with classifications of “yes” or “no.” Self-reported health questionnaires provided the data on diabetes and hypertension for each participant.

### 2.5. Statistical analysis

To ensure the representativeness of the sample in the general population, we used fasting subsample weights for weighted analysis, following the guidelines on the NHANES official website. Continuous variables are expressed as weighted means with corresponding standard errors, whereas categorical variables are expressed as weighted percentages. We deployed weighted logistic regression models to probe the associations between diverse TyG indices and OAB. Model 1 was baseline with no adjustments. Model 2 made adjustments for age, race/ethnicity, and sex. Model 3 was further refined to include marital status, PIR, educational levels, smoking status, hypertension, and diabetes, augmenting the prior adjustments in model 2. We report the results as odds ratios (ORs) with 95% confidence intervals (CIs). Restricted cubic splines were used to elucidate potential nonlinear interactions between the TyG indices and OAB, adjusted for confounders as specified in model 3. Subsequently, we undertook subgroup analyses and interaction tests for various categorical covariates, including gender, educational levels, race/ethnicity, marital status, smoking status, PIR, diabetes, and hypertension. To assess the robustness of our findings, we performed several sensitivity analyses: excluding participants with diabetes, excluding participants with hypertension, and excluding participants with extreme laboratory values. The discriminative performance of TyG, TyG-BMI, TyG-WC, and TyG-WHtR in identifying OAB was determined by reviewing receiver operating characteristic curves and computing the area under the curve (AUC). All procedures were carried out using R (version 4.3.0; R Foundation for Statistical Computing, Vienna, Austria) and RStudio (Posit Software, PBC, Boston), with significance determined at *P* < .05.

## 3. Results

### 3.1. Characteristics of the participants

Table 1 presents the baseline characteristics of the participants with and without OAB. Overall, among the total participants (N = 7841), 1962 individuals had OAB. There were significant differences (*P* < .05) between the control group and the OAB group in terms of age, sex, PIR, BMI, smoking status, race/ethnicity, marital status, education level, hypertension, diabetes, WHtR, height, waist circumference, fasting glucose, and different TyG indices. It was observed that participants with OAB were generally older, with a higher proportion of males, among other demographic characteristics.

**Table 1:** Characteristics of participants.

Characteristics	Overall (N = 7841)	Nonoveractive bladder (N = 5879)	Overactive bladder (N = 1962)	*P* value
Age (yr), mean ± SD	47.75 (17.02)	45.27 (16.37)	57.79 (15.87)	<.001
Gender, n (%)				<.001
Female	3845 (49.0%)	3083 (52.3%)	762 (35.6%)	
Male	3996 (51.0%)	2796 (47.7%)	1200 (64.4%)	
PIR, n (%)				<.001
<1.3	2099 (19.1%)	1469 (17.7%)	630 (24.4%)	
1.3–3.0	3098 (34.8%)	2248 (33.7%)	850 (39.2%)	
>3.0	2644 (46.2%)	2162 (48.6%)	482 (36.3%)	
BMI (kg/m^2^), mean ± SD	29.52 (7.17)	28.98 (6.77)	31.76 (8.23)	<.001
Smoked ≥ 100 cigarettes, n (%)				.008
Yes	3456 (44.4%)	2481 (43.2%)	975 (49.6%)	
No	4378 (55.6%)	3395 (56.8%)	983 (50.4%)	
Race/ethnicity, n (%)				<.001
Mexican American	1099 (8.9%)	844 (9.3%)	255 (7.7%)	
Non-Hispanic White	1713 (11.0%)	1139 (9.7%)	574 (16.4%)	
Non-Hispanic Black	2056 (15.4%)	1642 (15.9%)	414 (13.2%)	
Other	2973 (64.6%)	2254 (65.1%)	719 (62.7%)	
Marital status, n (%)				<.001
Married/living with partner	4712 (63.3%)	3641 (64.4%)	1071 (59.0%)	
Widowed/divorced/separated	2349 (26.7%)	1585 (24.7%)	764 (34.6%)	
Never married	776 (10.0%)	652 (10.9%)	124 (6.4%)	
Education level, n (%)				<.001
<High school	636 (4.2%)	414 (3.6%)	222 (6.3%)	
Completed high school	959 (8.8%)	650 (8.0%)	309 (12.2%)	
>High school	6244 (87.0%)	4815 (88.4%)	1429 (81.4%)	
Diabetes, n (%)				<.001
Yes	1141 (11.0%)	624 (8.5%)	517 (21.1%)	
No	6466 (89.0%)	5093 (91.5%)	1313 (78.9%)	
Hypertension, n (%)				<.001
Yes	2975 (33.0%)	1853 (28.6%)	1122 (50.5%)	
No	4857 (67.0%)	4017 (71.4%)	840 (49.5%)	
WHtR, mean ± SD	0.60 (0.10)	0.59 (0.10)	0.64 (0.11)	<.001
Waistline (cm), mean ± SD	100.50 (17.01)	99.09 (16.48)	106.21 (17.90)	<.001
Stand height (cm), mean ± SD	168.49 (9.88)	169.26 (9.81)	165.36 (9.52)	<.001
Fasting glucose (mg/dL), mean ± SD	108.32 (31.74)	106.60 (29.10)	115.32 (40.02)	<.001
Fasting triglyceride (mg/dL), mean ± SD	114.42 (100.32)	113.57 (103.62)	117.90 (85.52)	.138
TyG, mean ± SD	8.51 (0.69)	8.49 (0.68)	8.62 (0.68)	<.001
TyG-BMI, mean ± SD	252.76 (70.31)	247.32 (66.89)	274.91 (79.03)	<.001
TyG-WC, mean ± SD	859.77 (183.18)	845.24 (178.55)	918.86 (189.80)	<.001
TyG-WHtR, mean ± SD	5.11 (1.09)	5.00 (1.04)	5.57 (1.15)	<.001

BMI = body mass index, PIR = poverty-income ratio, SD = Standard Deviation, TyG-BMI = triglyceride-glucose body mass index, TyG-WC = triglyceride-glucose waist circumference, TyG-WHtR = triglyceride-glucose waist-to-height ratio, WHtR = waist-to-height ratio.

### 3.2. Associations between TyG-related indicators and OAB

**Figure 2.**
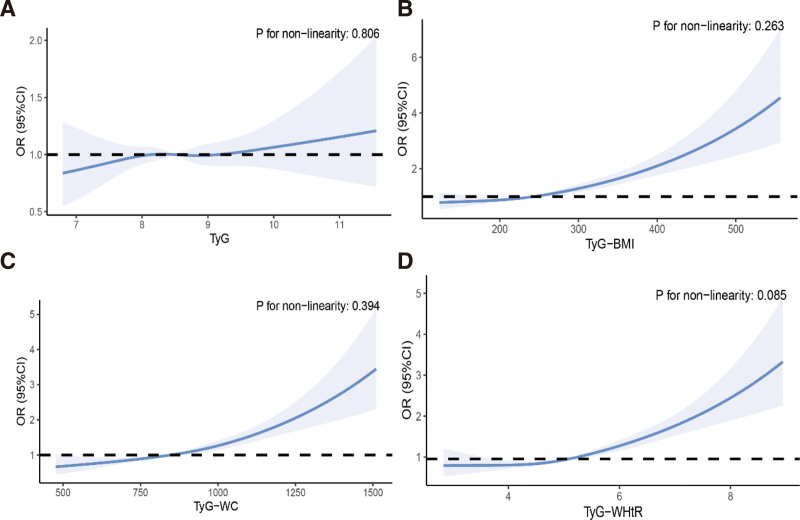
Restricted cubic spline fitting for the associations between TyG-related indices and OAB in model 3. (A) Restricted cubic spline fitting for the association between different TyG with OAB in model 3. (B) Restricted cubic spline fitting for the association between different TyG-BMI with OAB in model 3. (C) Restricted cubic spline fitting for the association between different TyG-WC with OAB in model 3. (D) Restricted cubic spline fitting for the association between different TyG-WHtR with OAB in model 3. CI = confidence interval, OAB = overactive bladder, OR = odds ratio, TyG = triglyceride-glucose, TyG-BMI = triglyceride-glucose body mass index, TyG-WC = triglyceride-glucose waist circumference, TyG-WHtR = triglyceride-glucose waist-to-height ratio.

A weighted multivariable logistic regression analysis was employed to further explore the relationship between OAB and TyG-related indices. Table 2 outlines the ORs and 95% CIs, explaining the associations between these indicators and OAB. Upon analyzing these associations using 3 varied models, it was determined that TyG-BMI, TyG-WC, and TyG-WHtR were independently and positively associated with OAB across all models. In model 3, TyG-WHtR showed a more significant association with OAB (Q4 OR [95% CI] = 2.138 [1.635–2.797]). As covariates were adjusted, the association between TyG and OAB became attenuated. Sensitivity analyses excluding participants with diabetes, hypertension, or extreme laboratory values yielded consistent results, confirming the robustness of our findings (see Tables S1–S3, Supplemental Digital Content, https://links.lww.com/MD/R534). Subsequent restricted cubic spline analysis (Fig. 2) validated a consistent positive linear relationship for TyG-BMI, TyG-WC, and TyG-WHtR with OAB across model 3 (*P* value < .05, nonlinearity > 0.05).

**Table 2:** Association between TyG-related indicators and OAB.

Exposures	OR (95% CI) *P*		
	Model 1	Model 2	Model 3
TyG			
Q1	Reference	Reference	Reference
Q2	1.139 (0.951–1.364) .153	0.942 (0.761–1.166) .577	0.922 (0.739–1.151) .466
Q3	1.375 (1.122–1.686) .003	1.128 (0.887–1.433) .318	1.023 (0.805–1.300) .850
Q4	1.679 (1.348–2.090) <.001	1.387 (1.061–1.813) .018	1.081 (0.803–1.455) .600
*P* for trend	<.001	.005	.518
TyG-WC			
Q1	Reference	Reference	Reference
Q2	1.486 (1.259–1.754) <.001	1.091 (0.875–1.361) .430	1.082 (0.863–1.357) .484
Q3	1.874 (1.490–2.357) <.001	1.453 (1.126–1.874) .005	1.360 (1.052–1.758) .020
Q4	2.750 (2.158–3.503) <.001	2.265 (1.718–2.987) <.001	2.006 (1.533–2.624) <.001
*P* for trend	<.001	<.001	<.001
TyG-WHtR			
Q1	Reference	Reference	Reference
Q2	1.748 (1.468–2.083) <.001	1.203 (0.986–1.467) .068	1.179 (0.953–1.459) .126
Q3	2.113 (1.699–2.626) <.001	1.434 (1.145–1.797) .002	1.314 (1.027–1.680) .031
Q4	3.958 (3.142–4.986) <.001	2.527 (1.959–3.261) <.001	2.138 (1.635–2.797) <.001
*P* for trend	<.001	<.001	<.001
TyG-BMI			
Q1	Reference	Reference	Reference
Q2	1.421 (1.190–1.695) <.001	1.123 (0.920–1.372) .246	1.143 (0.934–1.400) .189
Q3	1.673 (1.391–2.012) <.001	1.415 (1.143–1.750) .002	1.315 (1.049–1.648) .019
Q4	2.525 (2.008–3.173) <.001	2.220 (1.754–2.810) <.001	1.925 (1.498–2.472) <.001
*P* for trend	<.001	<.001	<.001

Model 1: unadjusted model.

Model 2: adjusted for age, race/ethnicity, and sex.

Model 3: further adjusted for marital status, PIR, educational levels, smoking status, hypertension, and diabetes.

CI = confidence interval, OAB = overactive bladder, OR = odds ratio, PIR = poverty-income ratio, TyG = triglyceride-glucose, TyG-BMI = triglyceride-glucose body mass index, TyG-WC = triglyceride-glucose waist circumference, TyG-WHtR = triglyceride-glucose waist-to-height ratio.

### 3.3. Subgroup analyses

**Figure 3.**
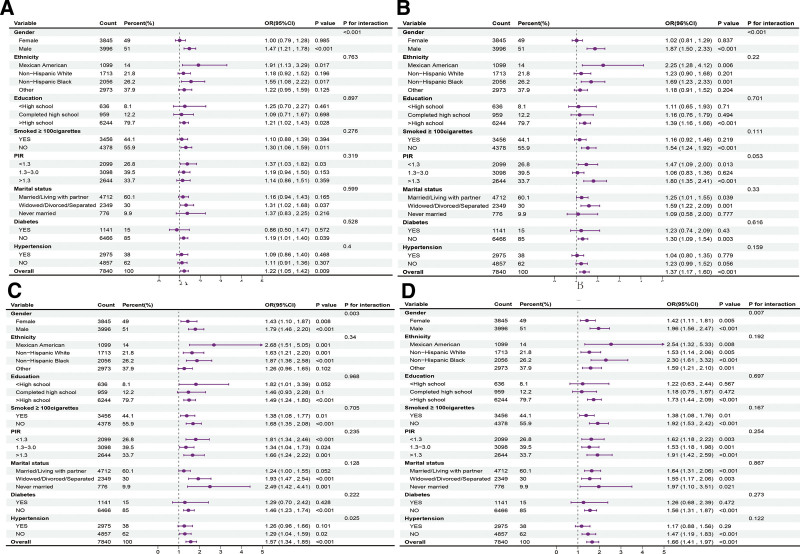
Forest maps of subgroup analysis on the relationship between TyG-related indices and OAB. (A) Forest maps of subgroup analysis on the relationship between TyG and OAB. (B) Forest maps of subgroup analysis on the relationship between TyG-BMI and OAB. (C) Forest maps of subgroup analysis on the relationship between TyG-WC and OAB. (D) Forest maps of subgroup analysis on the relationship between TyG-WHtR and OAB. CI = confidence interval, OAB = overactive bladder, OR = odds ratio, PIR = poverty-income ratio, TyG = triglyceride-glucose, TyG-BMI = triglyceride-glucose body mass index, TyG-WC = triglyceride-glucose waist circumference, TyG-WHtR = triglyceride-glucose waist-to-height ratio.

Subgroup analyses assessed the stability of the association between different TyG-related indices and OAB incidence. Tests were carried out to detect interactions based on sex, race/ethnicity, education level, smoking status, PIR, marital status, diabetes, and hypertension. Sex appeared as a significant interaction factor in the association between all improved TyG indices and OAB incidence (*P* < .05), with a stronger association noted in males (Fig. 3). Additionally, there was an interaction between TyG-WC and OAB incidence with hypertension history. In individuals without hypertension, the OR and 95% CI for TyG-WC were 1.29 (1.04, 1.59), respectively. Given that subgroup analyses involve multiple comparisons, which may inflate the type I error rate, these analyses should be considered exploratory in nature, and the interaction results should be interpreted with caution.

### 3.4. Comparison of different TyG-related indicators in identifying OAB

**Figure 4.**
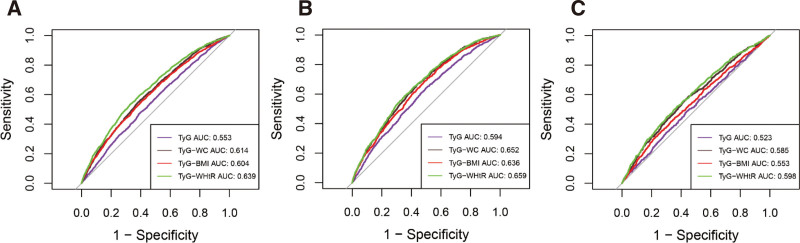
ROC curve analysis for predicting overactive bladder across different populations. (A) ROC curve analysis for predicting overactive bladder in the general population. (B) ROC curve analysis for predicting overactive bladder in the male population. (C) ROC curve analysis for predicting overactive bladder in the female population. AUC = area under the curve, ROC = receiver operating characteristic, TyG = triglyceride-glucose, TyG-BMI = triglyceride-glucose body mass index, TyG-WC = triglyceride-glucose waist circumference, TyG-WHtR = triglyceride-glucose waist-to-height ratio.

To better compare the discriminative ability of different TyG-related indices for identifying OAB, we calculated the AUC. The receiver operating characteristic curve results are shown in Figure 4. All improved TyG indices showed improved discriminative performance compared with the original TyG index. The TyG-WHtR, in particular, reached the highest AUC of 0.639. In both male and female subgroups, the AUC for any indicator was higher in males, suggesting that males are more susceptible to changes in TyG-related indices.

## 4. Discussion

Our research is the first of its kind, a nationally representative cross-sectional study that examines the association between TyG-related indices and the risk of OAB. After adjusting for potential confounders, the positive association between TyG-BMI, TyG-WC, TyG-WHtR, and OAB remained significant. In measuring sensitivity, TyG-BMI, TyG-WC, and TyG-WHtR also demonstrated stronger associations with OAB than the TyG index alone, highlighting their potential clinical relevance in understanding the metabolic aspects of OAB.

OAB, being a multifactorial and multimechanism disorder, often has underlying pathologies that are difficult to pinpoint in most patients. Recently, metabolic abnormalities, including dyslipidemia and impaired glucose metabolism, have been observed to be associated with OAB.^[7–10,19]^ As a reliable surrogate marker for IR, the TyG index has been validated in both diabetic and nondiabetic populations.^[20]^ In addition, indicators such as BMI, waist circumference, and WHtR are utilized as predictors of obesity and IR.^[21]^ The recent introduction of indices such as TyG-BMI, TyG-WC, and TyG-WHtR has resulted from integrating the TyG index with anthropometric measurements. These new indices incorporate abnormal glucose metabolism, defective fatty acid metabolism, and obesity metrics, making them potentially more reliable than the TyG index alone.^[22]^ This explains why our study observed that the combined indices have higher discriminative ability for identifying OAB compared with the TyG index alone.

Notably, IR stands as a significant indicator for conditions such as obesity, hypertension, dyslipidemia, and other MS. Studies have shown that in women, obesity greatly increases the risk of developing OAB.^[23]^ Other research has found a positive correlation between anthropometric measures such as the Body Roundness Index and the risk of OAB, particularly noting that men are more sensitive to this measure, which aligns with our study’s findings.^[24]^ Contemporary studies propose that MS is crucial in advancing OAB.^[7]^ OAB linked to MS could potentially be categorized as a specific subtype.^[25]^ Although direct evidence linking IR with OAB is scarce, an association between MS and OAB is possible. Researchers have observed that IR in the bladder mucosa can directly impair bladder function under metabolic conditions: in obese mouse models, insulin action defects in the bladder mucosa have been shown to impair detrusor muscle relaxation and lead to OAB.^[26]^ Additionally, IR can cause sympathetic overactivity^[27]^; previous studies have demonstrated that patients with OAB exhibit higher sympathetic nerve activity,^[28]^ which increases sensitivity to bladder filling, urgency, and incontinence. Moreover, research has observed that animal models of IR or diabetes exhibit reduced levels of nerve growth factor,^[29]^ and disruptions in nerve growth factor can affect the central nervous system’s control over urine storage and release.^[30]^ Chronic bladder ischemia is another proposed etiology for OAB.^[31]^ Known effects of IR include endothelial dysfunction, a critical factor in the development of atherosclerosis,^[32]^ which in turn leads to reduced bladder blood flow.

Interestingly, our study found that the male population is more affected by changes in TyG-related indices. The findings suggest that in the early identification of OAB, men may receive more significant benefits from TyG-related indices than women. We hypothesize that this may be due to the additional impact of prostate enlargement in men. Studies have shown that hyperinsulinemia and lipotoxicity can promote the growth of prostate tissue.^[33]^ The overlap of symptoms between OAB and benign prostatic hyperplasia complicates the diagnosis and treatment of OAB in men.^[34]^

This research benefits from the employment of a population-based dataset sourced from NHANES, coupled with suitable adjustments for covariates. The analysis also accounted for sampling weights, enhancing the reliability and statistical inference of the findings. Our analysis examined the individual associations of TyG-related indices with OAB, suggesting their potential value in understanding the metabolic factors associated with OAB. Furthermore, the assessment of IR through TyG-related indices is conducted using routine clinical care measurements, which dispenses with the need for specific insulin tests or hyperinsulinemic-euglycemic clamp. As a result, it becomes a readily accessible and reliable metric for epidemiological studies with clinical applications. However, several constraints of this study must be acknowledged. The foremost is that its cross-sectional nature impedes the establishment of causality between TyG-related indices and OAB. Second, OAB in this study was defined using an OABSS score ≥ 3, which differs from the ICS clinical definition that relies on the presence of urgency, with or without urinary frequency and nocturia, without a specific numerical cutoff. This discrepancy between the questionnaire-based definition and the clinical diagnostic criteria may introduce potential misclassification of OAB status. Third, several key factors potentially associated with both metabolic health and OAB, such as physical activity, medications affecting bladder function, menopausal status, and LUTS/benign prostatic hyperplasia, were not available in our analysis, which may result in residual confounding. Finally, the confinement of the participant pool to the US population narrows the scope of our study’s external validity. Hence, the generalizability of these findings to other international populations may be restricted. Longitudinal and prospective research, together with exhaustive data collection, is indispensable for confirming the associations between TyG indices and OAB.

## 5. Conclusion

Our findings suggest that elevated TyG-BMI, TyG-WC, and TyG-WHtR levels are associated with OAB. This underscores their potential to serve as accessible indicators associated with OAB, warranting further investigation. Additional prospective and longitudinal studies are required to confirm these associations and dissect the underlying mechanisms.

## Acknowledgments

We would like to thank the publicly available data from the NHANES study.

## Author contributions

**Conceptualization:** Jifang Yang, Dejian Hou.

**Methodology:** Yong Luo.

**Formal analysis:** Yong Luo.

**Software:** Yong Luo, Dejian Hou.

**Data curation:** Shurui Li.

**Investigation:** Shurui Li, Yi Lu.

**Supervision:** Jifang Yang.

**Visualization:** Dejian Hou.

**Writing – original draft:** Yong Luo.

**Writing – review & editing:** Yong Luo, Shurui Li, Jifang Yang, Yi Lu, Dejian Hou.

## Supplementary Material
