# Supplementary material for: Positive association between different triglyceride-glucose index-related indicators and overactive bladder: Results from NHANES (2013–2020)
Source: Medicine (Baltimore). 2026 Mar 13;105(11):e47949. doi: 10.1097/MD.0000000000047949 (PMC12991677; doi:10.1097/MD.0000000000047949)
Supplement: Supplementary file 1 [file medi-105-e47949-s001.docx]

**Supplementary Table 1** Association between TyG-related indices and OAB after excluding participants with diabetes

| Exposures | OR (95% CI) *P* | | |
| --- | --- | --- | --- |
|  | Model 1 | Model 2 | Model 3 |
| TyG | | | |
| Q1 | Reference | Reference | Reference |
| Q2 | 1.094 (0.900-1.329) 0.361 | 0.926 (0.737-1.163) 0.501 | 0.881 (0.691-1.122) 0.296 |
| Q3 | 1.331 (1.066-1.663) 0.013 | 1.149 (0.872-1.515) 0.315 | 1.079 (0.813-1.432) 0.588 |
| Q4 | 1.374 (1.081-1.748) 0.011 | 1.285 (0.959-1.720) 0.091 | 1.143 (0.829-1.575) 0.404 |
| *P* for trend | 0.015 | 0.013 | 0.051 |
| TyG-WC |  |  |  |
| Q1 | Reference | Reference | Reference |
| Q2 | 1.403 (1.177-1.673) <0.001 | 1.049 (0.834-1.320) 0.677 | 1.015 (0.795-1.295) 0.905 |
| Q3 | 1.695 (1.317-2.180) <0.001 | 1.400 (1.057-1.856) 0.020 | 1.336 (0.993-1.797) 0.056 |
| Q4 | 2.358 (1.828-3.043) <0.001 | 2.144 (1.617-2.844) <0.001 | 1.951 (1.432-2.659) <0.001 |
| *P* for trend | <0.001 | <0.001 | <0.001 |
| TyG-WHtR |  |  |  |
| Q1 | Reference | Reference | Reference |
| Q2 | 1.653 (1.343-2.034) <0.001 | 1.160 (0.917-1.468) 0.210 | 1.134 (0.885-1.453) 0.312 |
| Q3 | 2.010 (1.572-2.570) <0.001 | 1.425 (1.111-1.828) 0.006 | 1.356 (1.034-1.777) 0.028 |
| Q4 | 3.441 (2.669-4.436) <0.001 | 2.383 (1.810-3.137) <0.001 | 2.150 (1.595-2.897) <0.001 |
| *P* for trend | <0.001 | <0.001 | <0.001 |
| TyG-BMI |  |  |  |
| Q1 | Reference | Reference | Reference |
| Q2 | 1.347 (1.093-1.661) 0.006 | 1.085 (0.861-1.367) 0.519 | 1.077 (0.854-1.359) 0.519 |
| Q3 | 1.559 (1.250-1.945) <0.001 | 1.419 (1.107-1.819) 0.025 | 1.358 (1.042-1.769) 0.025 |
| Q4 | 2.148 (1.681-2.745) <0.001 | 2.030 (1.596-2.581) <0.001 | 1.908 (1.457-2.498) <0.001 |
| *P* for trend | <0.001 | <0.001 | <0.001 |
| Model 1: unadjusted model. | | | |
| Model 2: adjusted for age, race/ethnicity, and sex. | | | |
| Model 3: further adjusted for marital status, PIR, educational levels, smoking status and hypertension. | | | |
|  |  |  |  |
|  |  |  |  |

**Supplementary Table 2** Association between TyG-related Indicators and OAB after excluding participants with hypertension

| Exposures | OR (95% CI) *P* | | |
| --- | --- | --- | --- |
|  | Model 1 | Model 2 | Model 3 |
| TyG | | | |
| Q1 | Reference | Reference | Reference |
| Q2 | 1.190 (0.892-1.589) 0.232 | 1.061 (0.779-1.444) 0.702 | 1.000 (0.722-1.386) 0.998 |
| Q3 | 1.204 (0.955-1.518) 0.114 | 1.098 (0.825-1.462) 0.513 | 1.015 (0.755-1.365) 0.919 |
| Q4 | 1.766 (1.337-2.332) <0.001 | 1.731 (1.225-2.445) 0.003 | 1.402 (0.938-2.095) 0.097 |
| *P* for trend | <0.001 | 0.005 | 0.182 |
| TyG-WC |  |  |  |
| Q1 | Reference | Reference | Reference |
| Q2 | 1.325 (1.003-1.749) 0.048 | 1.046 (0.768-1.424) 0.773 | 0.981 (0.707-1.361) 0.906 |
| Q3 | 1.983 (1.545-2.545) <0.001 | 1.710 (1.269-2.302) <0.001 | 1.555 (1.125-2.151) 0.009 |
| Q4 | 2.548 (2.008-3.234) <0.001 | 2.358 (1.782-3.120) <0.001 | 1.983 (1.532-2.568) <0.001 |
| *P* for trend | <0.001 | <0.001 | <0.001 |
| TyG-WHtR |  |  |  |
| Q1 | Reference | Reference | Reference |
| Q2 | 1.598 (1.190-2.145) 0.002 | 1.260 (0.916-1.732) 0.151 | 1.198 (0.849-1.691) 0.295 |
| Q3 | 2.171 (1.710-2.756) <0.001 | 1.712 (1.344-2.181) <0.001 | 1.567 (1.186-2.068) 0.002 |
| Q4 | 3.540 (2.707-4.630) <0.001 | 2.691 (2.003-3.617) <0.001 | 2.245 (1.684-2.992) <0.001 |
| *P* for trend | <0.001 | <0.001 | <0.001 |
| TyG-BMI |  |  |  |
| Q1 | Reference | Reference | Reference |
| Q2 | 1.429 (1.039-1.965) 0.006 | 1.214 (0.857-1.718) 0.268 | 1.209 (0.851-1.717) 0.281 |
| Q3 | 1.771 (1.407-2.231) <0.001 | 1.707 (1.308-2.228) <0.001 | 1.581 (1.194-2.095) 0.002 |
| Q4 | 2.420 (1.883-3.111) <0.001 | 2.353 (1.798-3.081) <0.001 | 2.041 (1.561-2.669) <0.001 |
| *P* for trend | <0.001 | <0.001 | <0.001 |
| Model 1: unadjusted model. | | | |
| Model 2: adjusted for age, race/ethnicity, and sex. | | | |
| Model 3: further adjusted for marital status, PIR, educational levels, smoking status and diabetes | | | |
|  |  |  |  |
|  |  |  |  |

**Supplementary Table 3** Association between TyG-related indices and OAB excluding extreme laboratory values (<2.5th or >97.5th percentile)

| Exposures | OR (95% CI) *P* | | |
| --- | --- | --- | --- |
|  | Model 1 | Model 2 | Model 3 |
| TyG | | | |
| Q1 | Reference | Reference | Reference |
| Q2 | 1.142 (0.951-1.372) 0.150 | 0.954 (0.768-1.187) 0.669 | 0.909 (0.726-1.139) 0.398 |
| Q3 | 1.379 (1.123-1.693) 0.003 | 1.160 (0.907-1.484) 0.231 | 1.030 (0.797-1.333) 0.814 |
| Q4 | 1.630 (1.295-2.051) <0.001 | 1.325 (1.005-1.747) 0.047 | 1.021 (0.754-1.382) 0.892 |
| *P* for trend | <0.001 | 0.043 | 0.615 |
| TyG-WC |  |  |  |
| Q1 | Reference | Reference | Reference |
| Q2 | 1.474 (1.228-1.768) <0.001 | 1.107 (0.876-1.398) 0.386 | 1.066 (0.835-1.361) 0.598 |
| Q3 | 1.875 (1.486-2.368) <0.001 | 1.490 (1.152-1.927) 0.003 | 1.337 (1.023-1.748) 0.034 |
| Q4 | 2.721 (2.116-3.499) <0.001 | 2.249 (1.684-3.003) <0.001 | 1.843 (1.368-2.483) <0.001 |
| *P* for trend | <0.001 | <0.001 | <0.001 |
| TyG-WHtR |  |  |  |
| Q1 | Reference | Reference | Reference |
| Q2 | 1.797 (1.498-2.156) <0.001 | 1.227 (1.001-1.505) 0.049 | 1.178 (0.947-1.466) 0.137 |
| Q3 | 2.136 (1.690-2.699) <0.001 | 1.441 (1.124-1.846) 0.005 | 1.284 (0.980-1.684) 0.069 |
| Q4 | 3.964 (3.143-4.999) <0.001 | 2.511 (1.944-3.242) <0.001 | 2.030 (1.551-2.657) <0.001 |
| *P* for trend | <0.001 | <0.001 | <0.001 |
| TyG-BMI |  |  |  |
| Q1 | Reference | Reference | Reference |
| Q2 | 1.413 (1.199-1.665) <0.001 | 1.144 (0.952-1.375) 0.149 | 1.161 (0.961-1.402) 0.118 |
| Q3 | 1.614 (1.331-1.958) <0.001 | 1.390 (1.117-1.729) 0.004 | 1.265 (0.996-1.606) 0.053 |
| Q4 | 2.493 (1.983-3.134) <0.001 | 2.236 (1.773-2.819) <0.001 | 1.912 (1.485-2.462) <0.001 |
| *P* for trend | <0.001 | <0.001 | <0.001 |
| Model 1: unadjusted model. | | | |
| Model 2: adjusted for age, race/ethnicity, and sex. | | | |
| Model 3: further adjusted for marital status, PIR, educational levels, smoking status, hypertension and diabetes | | | |
|  |  |  |  |
|  |  |  |  |
